# Supplementary material for: Hydrophobin CmHYD1 Is Involved in Conidiation, Infection and Primordium Formation, and Regulated by GATA Transcription Factor CmAreA in Edible Fungus, Cordyceps militaris
Source: J Fungi (Basel). 2021 Aug 20;7(8):674. doi: 10.3390/jof7080674 (PMC8400498; doi:10.3390/jof7080674)
Supplement: Supplementary file 1 [file jof-07-00674-s001.zip › jof-1340022-supplementary.pdf]

Journal of
Fungi

MDPI

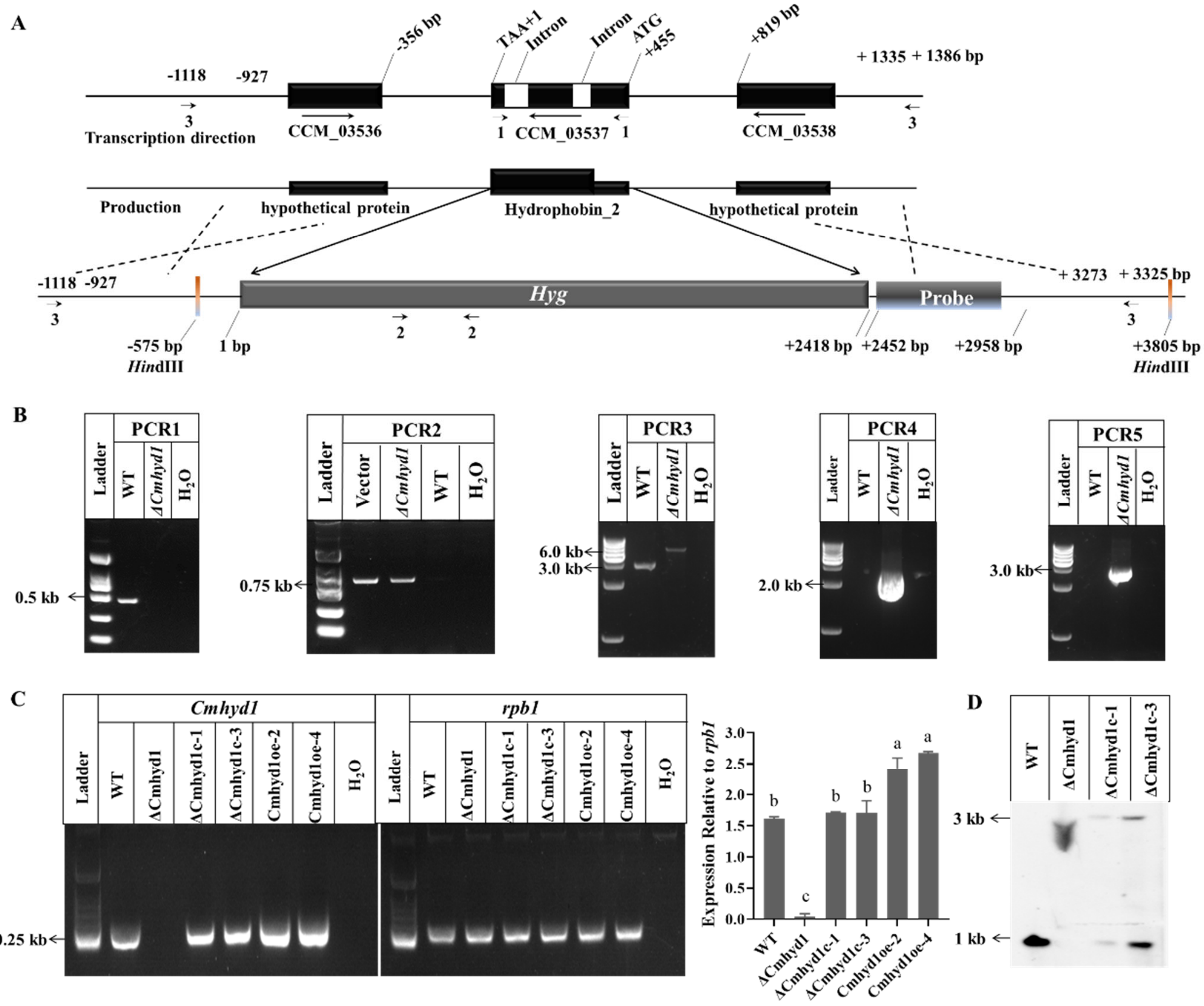

Figure S1. Generation and identification of *Cmhyd1* mutants in *Cordyceps militaris*. (**A**) Schematic diagram showing the generation of *ΔCmhyd1 via* homologous recombination and southern blot analysis. Bases are numbered starting with the translational initiation codon for *Cmhyd1* (CCM_03537). Hydrophobin_2 was the functional domains in CmHYD1. CCM_03536 and CCM_03538 are the up- and downstream-genes of *Cmhyd1*, respectively. *Hyg* is the hygromycin resistance gene. Probe is for the southern blot hybridization. The primers 1 (Cmhyd1-verti-F/ Cmhyd1-verti-R), 2 (Hyg-F/Hyg-R), and 3 (Cmhyd1-up-F/ Cmhyd1-down-R) were for identification. (B) Confirmation of *ΔCmhyd1* by PCR. PCR1, PCR2, and PCR3 were performed with the primers 1, 2 and 3 respectively. PCR4 and PCR5 were performed with the primers 3F/2R and 2F/3R respectively. WT, the wild-type strain. DNA fragments with lengths of 421, 800, 4443, 2287 and 2956 bp were obtained using primer sets 1, 2, 3, 3F/2R and 2F/3R from the *ΔCmhyd1* strain, respectively. (**C**) The transcript levels of *Cmhyd1* in the WT and *Cmhyd1* mutants by RT-PCR. Total RNA extraction and cDNA synthesis were performed as described in the experimental procedures. Transcript levels of *Cmhyd1* and *rpb1* were detected with the primers qCmhyd1-F/qCmhyd1-R and qrpb1-F/qrpb1-R, respectively. The expression levels of *Cmhyd1* relative to *rpb1* were presented by optical density ratio using ImageJ. (**D**) Southern blot assay validating the *Cmhyd1* gene deletion and complementation strains. Lane 1: WT, Lanes 2: *ΔCmhyd1* strains, Lane 3 and 4: *ΔCmhyd1* complementation strain. Genomic DNAs were digested with *Hin*dIII/*Hin*dIII for probing *Cmhyd1*.

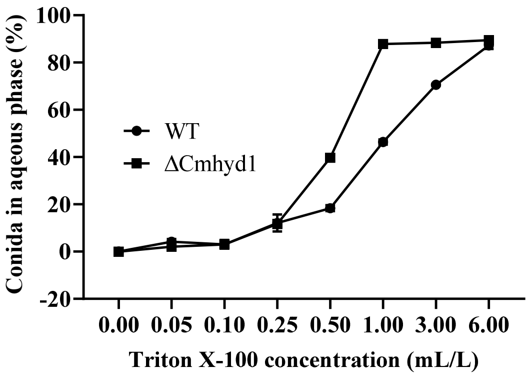

Figure S2. Hydrophobicity of the conidia determined using a MATH assay. Conidia of WT (●) and *ΔCmhyd1* (■) strains were suspended in PBS/hexadecane mixture and shaken vigorously. The amount of conidia in the aqueous phase in the presence of various amounts of Triton X-100 was shown (each point was the mean of three replicates). The more hydrophobic conidia required higher concentrations of detergent to release them from the oil-water interface.

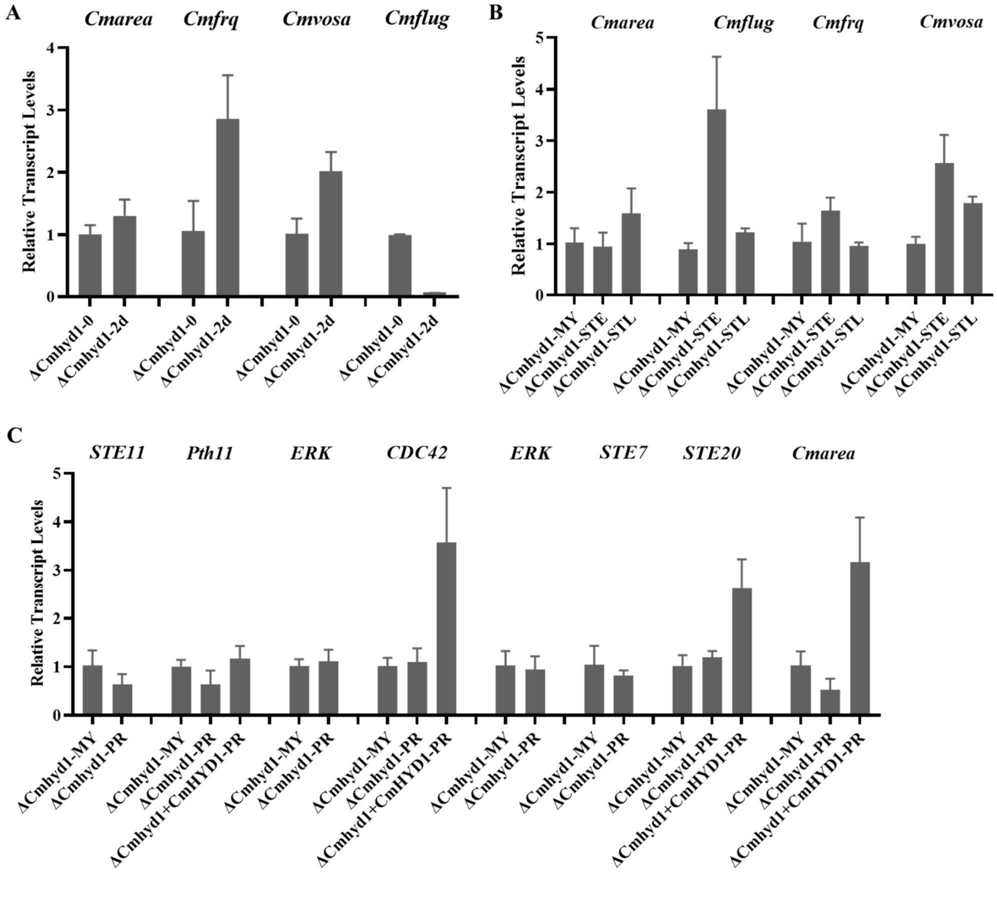

Figure S3. Relative transcript levels in the *ΔCmhyd1* strain. (**A**) The relative transcript levels of *Cmhyd1* and conidiation-related genes after being exposed to light for 2 d. All results were based on the standard levels of darkness stage of the *ΔCmhyd1* strain (ΔCmhyd1-0). (**B**) The relative transcript levels of *Cmhyd1* and infection-related genes at the different cuticle-bypassing infection stages. All results were based on the standard levels of the MY stage of *ΔCmhyd1* strain (ΔCmhyd1-MY). (**C**) The relative transcript levels of *Cmarea* and fruiting body development related genes at the primordium stage. All results were based on the standard levels of the MY stage of *ΔCmhyd1* strain (ΔCmhyd1-MY). Error bars indicate the standard deviation (SD) of three biological replicates with two technical replicates.

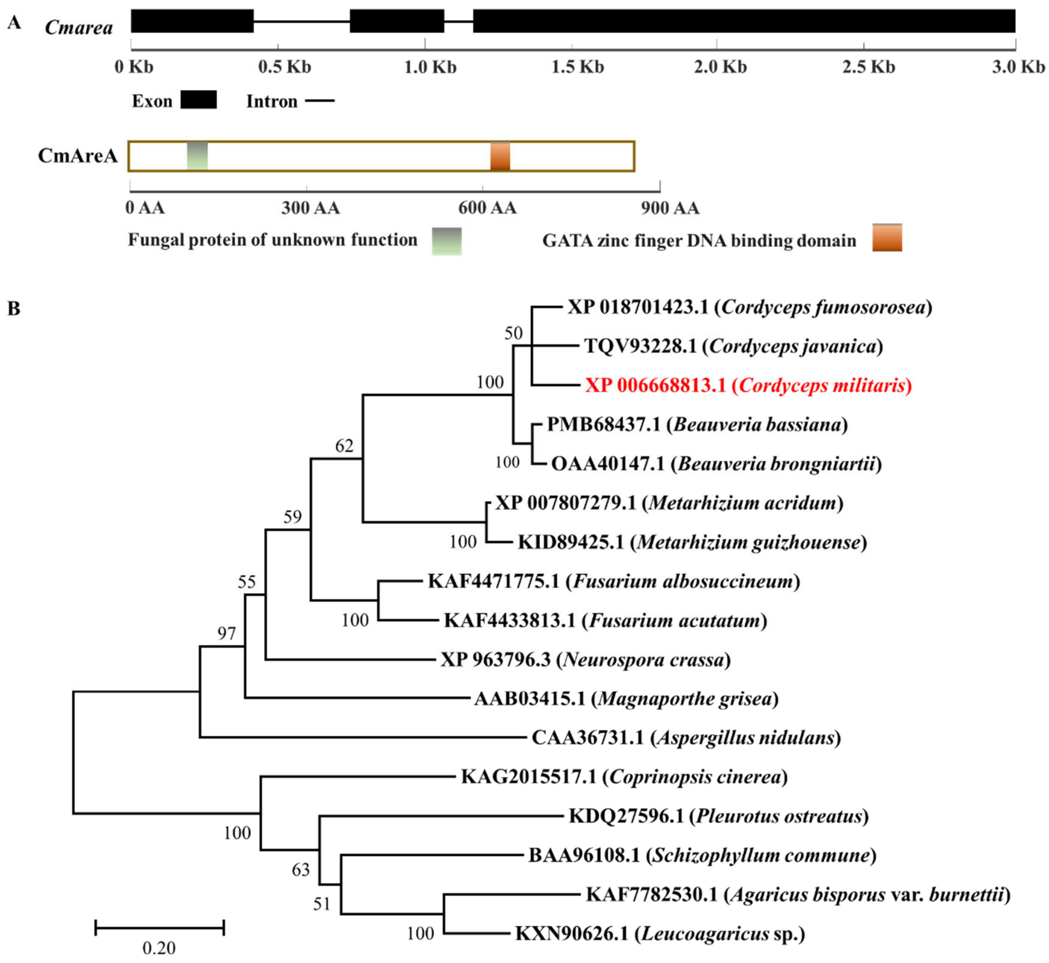

Figure S4. Characterization of the GATA zinc finger (ZnF) transcript factor gene *Cmarea* in *Cordyceps militaris*. (**A**) The gene structure and protein domains of CmAreA. (**B**) Phylogenetic relationships of CmAreA (red colored) and its homologs. The amino acid sequences were aligned with MEGAX, and a neighbor-joining tree was generated with 1000 bootstrap replicates using the program MEGAX.

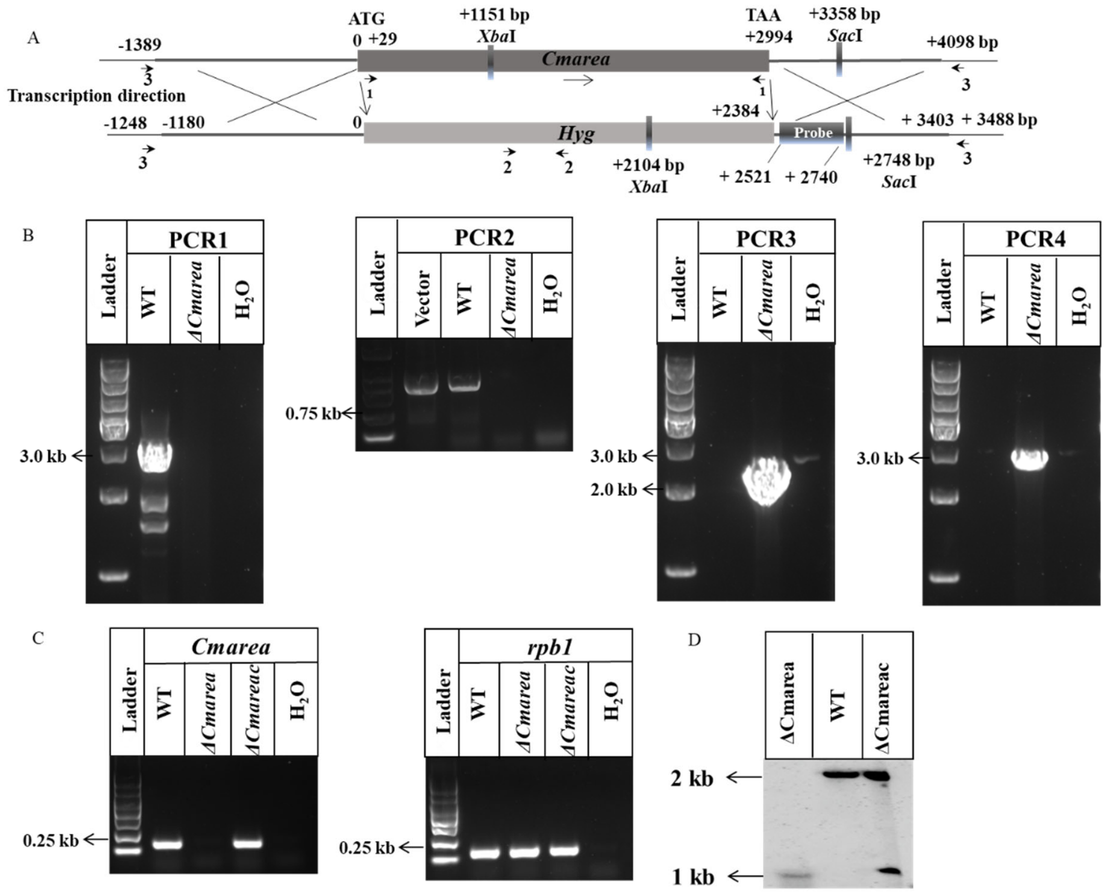

Figure S5. Generation and identification of *Cmarea* mutants in *Cordyceps militaris*. (**A**) Schematic diagram showing the generation of *ΔCmarea via* homologous recombination and southern blot analysis. (**B**) Confirmation of *ΔCmarea* by PCR. PCR1, PCR2, and PCR3 were performed with the primers 1, 2, 3F/2R and 2F/3R respectively. WT, the wild-type strain. DNA fragments with lengths of 2664, 800, 2417 and 3119 bp were obtained using primer sets 1, 2, 3F/2R and 2F/3R from the *ΔCmhyd1* strain respectively. (**C**) The transcript levels of *Cmarea* in the WT, *ΔCmarea* and *ΔCmareac* strains using RT-PCR. Total RNA extraction and cDNA synthesis were performed as described in the experimental procedures. Transcript levels of *Cmarea* and *rpb1* were detected with the primers qCmarea-F/qCmarea-R and qrpb1-F/qrpb1-R, respectively. (**D**) Southern blot assay validating the *Cmarea* gene deletion and complementary strains. Lane 1: *ΔCmarea* strain for 1254 bp, Lane 2: WT strain for 2207 bp, Lane 3: *ΔCmare* complementary strain for 1254 and 2207 bp. Genomic DNAs were digested with *Xba* Ⅰ/*Sac* Ⅰ for probing *Cmarea* in southern blot hybridization.

Table S1. Primers used in this study.

Primer Name	Primer Sequence (5' to 3')
rpb1-F	CTGTTCCCCCTCCTCCTGTG
rpb1-R	ATGTTGCGGCGATCCTTCTC
qPCR-*Cmhyd1*-F	CCTCTACTCCAACCCCATCTG
qPCR-*Cmhyd1*-R	AGGACTGCCTGGTCAATCAC
Cmhyd1-verti-F1	GCAAGTCCTTCTCATTGCTAC
Cmhyd1-verti-R1	GCCTGGTCAATCTGTTCAGATG
Hyg-verti-F2	CTGTCGAGAAGTTTCTGATCG
Hyg-verti-R2	CTGATAGAGTTGGTCAAGACC
Cmhyd1-verti-F3	GCTTTCTGCATAGTTTTCTCCATT

Cmhyd1-verti-R3	ACCAAGTGTACATTAATTGAAAGC
Sothern Blot-*Cmhyd1*-F	TGGGCTGTTCTTGTCCTGC
Sothern Blot-*Cmhyd1*-R	GGCGAAATGTAAACGGAGC
qPCR-*Cmarea*-F	TGCCATCCATCTCGTCCAC
qPCR-*Cmarea*-R	TGAGCCACTCCCATTCCTG
Cmarea-verti-F1	AACCAGCAACGCATGGAAAACCTC
Cmarea-verti-R1	TTACAGACTCATCGTGAGCCACTC
Cmarea-verti-F3	TTCCAACAGCCAGACAAACAAGAC
Cmarea-verti-R3	ATAGCCTTGTTGCTACGTTTACGAG
Sothern Blot-*Cmarea*-F	CTACTCTTGCGTGTTTCTTTTTATAC
Sothern Blot-*Cmarea*-R	GTAGTCCAAGGAGATTAAGCGC
Cmarea-verti-F	AACCAGCAACGCATGGAAAACCTC
Cmarea-verti-R	TTACAGACTCATCGTGAGCCACTC
Biotin probe 1	TTCGCCTTCTCACCATCCCAAGATACTCCCATCAGCCAAGATGCAA
Biotin probe 2	GAGGACAACGATCCTCTCCTTGATACTCACAGGCCTGACTTCAAAC
Unlable	CAGTGAGACGGGACCGGCATCGATAGAACGCTGCAGCCACTGCCAA
